# Characteristics of a 1200 V Hybrid Power Switch Comprising a Si IGBT and a SiC MOSFET †

**DOI:** 10.3390/mi15111337

**Published:** 2024-10-31

**Authors:** Alireza Sheikhan, E. M. Sankara Narayanan

**Affiliations:** Department of Electronic and Electrical Engineering, The University of Sheffield, Sheffield S1 3JD, UK; s.madathil@sheffield.ac.uk

**Keywords:** silicon carbide (SiC), silicon (Si), Hybrid Power Switch, IGBT, MOSFET

## Abstract

Hybrid Power Switches (HPS) combine the advantages of SiC unipolar and Si bipolar devices and therefore can bridge the gap between these technologies. In this paper, the performance of a hybrid power switch configuration based on the latest SiC MOSFET and Si IGBT technologies is presented. The device is evaluated through experimental measurements of its characteristics under various conditions. The results show the HPS can achieve switching losses as low as a SiC MOSFET while offering the high current capability of the IGBT without significant increase in costs.

## 1. Introduction

Silicon insulated gate bipolar transistors (IGBTs) have conquered medium power applications including motor drives and electric vehicles thanks to their excellent performance over cost ratio. IGBTs benefit from strong conductivity modulation to achieve on-state resistance below the one-dimensional (1D) material limit of silicon. However, IGBTs have long switching transients during turn-off as the excess carriers generated due to the conductivity modulation need to be removed, which contributes to increased switching power losses. Furthermore, Si IGBTs suffer from an inherent P–N junction voltage drop of ~0.7 V at room temperature, which hinders low current performance. Reverse current flow is also not supported in conventional field stop IGBTs which necessitates additional anti-parallel diodes. Meanwhile, FETs utilizing wide bandgap materials such as SiC have gained significant traction in recent years due to their superior material characteristics. 4H-SiC offers a wide bandgap of 3.23 eV and high critical electric field of 2.5 MV·cm^−1^. Therefore, for a given voltage rating, devices with thinner, more highly doped drift regions can be designed to result in a significant reduction in on-state resistance. Unlike their bipolar counterparts, FETs are unipolar devices where only one type of charge carrier contributes to current flow. Therefore, there is no excess carrier that needs to be removed or injected. For this reason, very high switching speed can be achieved with switching losses significantly lower than Si IGBTs [1]. The I–V performance, due to the absence of an inherent P–N junction voltage drop, is particularly preferred over Si-based IGBTs. FETs also support current flow in reverse direction, via the body diode, which eliminates the need for additional anti-parallel diodes. However, environmental and economical costs associated with crystal growth, fabrication, and processing of SiC have significantly hindered large scale adaptation of the technology.

In many applications, such as electric vehicles (EVs), the source of power is often constrained by weight, size, and energy density. Thus, maintaining high efficiency over a wide range of load conditions is critical for effective system performance. Currently, most EVs incorporate Si IGBT modules alongside SiC MOSFET modules in their powertrain to maximize performance and efficiency across various load conditions. From a performance over cost perspective, hybrid power switches can be promising for reasons of costs and design as well as reliability and redundancy. HPS considered herein are formed by a parallel configuration of bipolar and unipolar devices (typically an IGBT and a MOSFET). HPS combine the fast switching and low on-state resistance of MOSFETs with high current capability of IGBTs to handle loads with minimal power loss. One of the early demonstrations of HPS was presented in 1983 [2]. To this date, several studies have reported various configurations of HPS, both as discrete and recently in module format [3,4,5,6,7,8,9,10,11,12,13,14]. The gate driver is particularly important for HPS optimum operation due to different switching characteristics between its constituent components. IGBTs generally have a longer delay time than MOSFETs. Several techniques have been reported which rely on imposing a delay (deadtime) in such a way that the switching transitions are controlled by the integral MOSFET. However, the selection of gate drive strategy has not been fully explored.

In this paper, a hybrid device configuration based on the latest Si IGBT and SiC MOSFET technologies is reported and analyzed. The characteristics, switching behavior, and short-circuit capability are experimentally demonstrated. Additionally, the impact of gate drive on switching performance is evaluated in relation to power losses.

## 2. Principle of Operation

The proposed HPS consists of a Si IGBT (IGBT7—IKW50N120CS7—Infineon [15]) and a SiC MOSFET (CoolSiC—IMW120R030M1H—Infineon [16]) in parallel arrangement. Both devices are rated for 1200 V and 50 A and housed in TO-247 packages. A soft recovery Si diode is also integrated in the same package as the IGBT. The active area ratio of the IGBT to the MOSFET equals to about 3:1.

**Figure 1:**
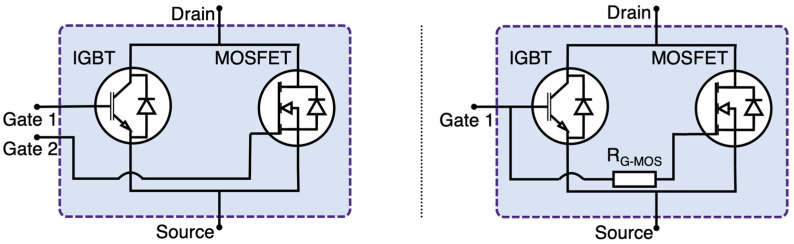
HPS dual gate configuration (**left**) and single gate configuration (**right**).

**Figure 2:**
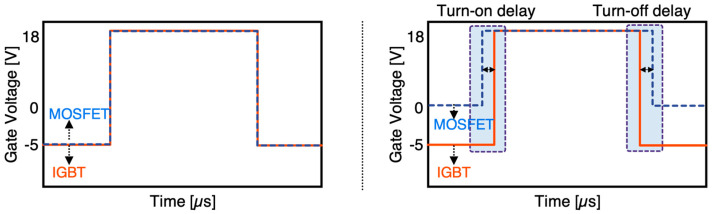
Typical gate control strategies for single gate (**left**) and dual gate (**right**) configurations.

As depicted in Figure 1, the HPS can be configured as a three-terminal device where gates are connected (single gate) or a four-terminal device where gates are separated (dual gate) and each can be controlled independently. Typical gate control strategies are presented in Figure 2.

The single gate configuration does not require additional gate drive components and is aimed for pin-for-pin compatibility with IGBTs. The single gate HPS offers superior performance compared with equivalent IGBT solutions [3]. However, the switching performance is not fully optimized. The dual gate configuration enables further optimization by implementing a delay between gate signals for each component in such a way that the MOSFET handles the entire load current in switching transitions. The delay is necessary particularly during turn-off switching to guarantee that the MOSFET turns-off after the IGBT. This is because IGBTs generally have longer switching times that can overlap with that of the MOSFET, leading to slower switching. During the delay period, the current is diverted through the MOSFET. In this method, the IGBT does not contribute to switching losses as it undergoes a zero-voltage switching. The turn-on delay, however, is not always necessary as the MOSFET naturally turns-on first due to its shorter delay time.

In terms of cost, implementation of HPS contributes to a lower overall outlay. This is because in target applications, multiple gate drivers are already utilized to drive IGBTs or SiC MOSFETs modules. Therefore, the gate drivers can be repurposed for the HPS with minimal alteration. The intelligent control can also be achieved through the use of existing microprocessor units, which are already used to derive the power stage. Meanwhile, the HPS reduces the burden on thermal management by offering superior efficiency.

## 3. Experimental Methodology

**Figure 3:**
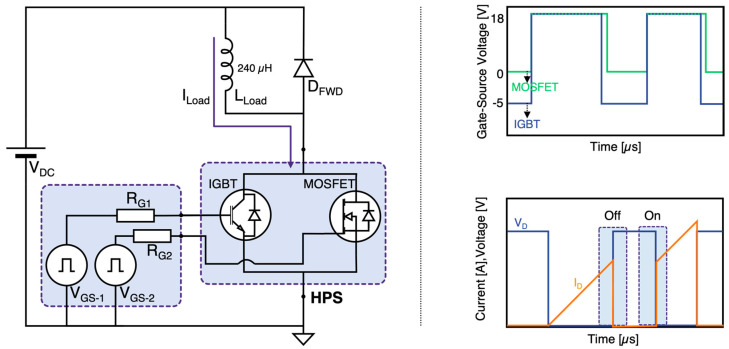
Experimental setup and typical switching waveforms.

The device characteristics were assessed by a number of tests. First the static characteristics were measured using a curve tracer to determine the I–V performance and device capacitances. Then, to evaluate the switching characteristics of the HPS, a double pulse clamped inductive test bench was set up as depicted in Figure 3.

Two independent gate drivers are used to drive the HPS dual gate configuration. Both gate drivers supply a gate bias of 18 V for turn-on. The turn-off gate bias is set to 0 V and −5 V for the constituent MOSFET and IGBT, respectively. In the single gate configuration, the applied gate voltages are 18 V and −5 V for turn-on and turn-off respectively. A 240 µH inductor is used as the load along with a 1200 V freewheeling SiC Schottky diode (D_FWD_) in parallel arrangement. In the dual gate HPS, the gate resistance of the IGBT (R_G1_) is fixed at 47 Ω and 15 Ω for turn-on and turn-off respectively. For the purpose of comparison, two paralleled MOSFETs (2×MOSFETs) and two paralleled IGBTs (2× IGBTs) were also tested under the same conditions as the HPS constituents. In case of the 2×MOSFETs a 10 Ω current sharing gate resistor was used to prevent gate oscillations. Based on the measurements, the switching time, slew rate, and dissipated energy were calculated. The switching time is defined as the change in switching voltage from 10% to 90%. The measurements were carried out at 25 °C unless stated otherwise. Finally, the short-circuit capability of the HPS was evaluated at elevated temperature for 3 µs. The gate voltage was set to 15 V based on the manufacturer recommendation.

## 4. Experimental Results and Discussion

### 4.1. Static Characteristics

#### 4.1.1. Current Voltage Characteristics

**Figure 4:**
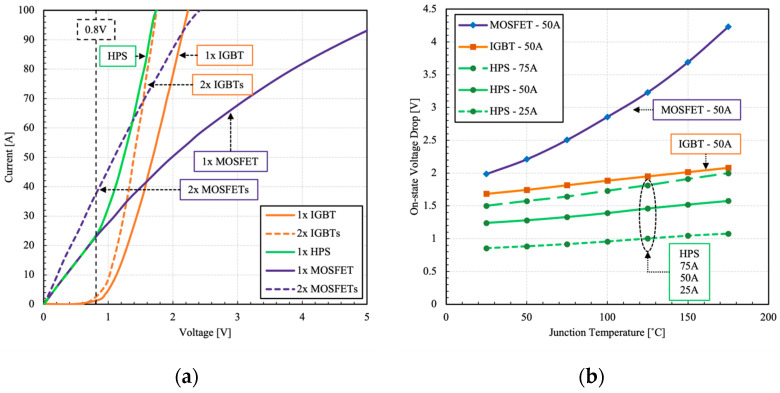
Measured (**a**) Output I–V characteristics at RT and (**b**) the corresponding on-state voltage drop of the HPS and its constituent components. The pulse width is 250 µs and V_GS_ is 18 V.

The on-state characteristics of the HPS and its constituents are shown in Figure 4.

The HPS benefits from a unique dual mode (bi-mode) operation, which means that it can operate either in unipolar or bipolar mode selectively. The boundary condition between unipolar and bipolar modes is governed by the on-state resistance of the MOSFET and the bipolar on-set voltage of the IGBT, which are both temperature dependent. In this HPS configuration, the boundary condition is 25 A at RT. Below this boundary, the device operates purely in unipolar mode. Beyond this point, the voltage drop across the device is high enough to overcome the bipolar on-set voltage of the IGBT (~0.7 V) and the device operates in bipolar mode. Consequently, the on-state resistance is reduced due to conductivity modulation, resulting in a lower voltage drop, seen as a steeper line in the I–V curves. In bipolar regime, the excess current is shared between the individual devices according to their on-state resistance values. The on-state voltage was extracted for each device from the measured I–V curves at different junction temperatures and currents, as shown in Figure 4b. The HPS maintains a mild positive coefficient of temperature of the on-state voltage under various load current conditions. The bipolar on-set voltage decreases with the rise of temperature, which helps to compensate for the increase in resistance. This feature enables the HPS to maintain a low conduction loss profile over a wide range of load currents and ambient temperatures.

**Figure 5:**
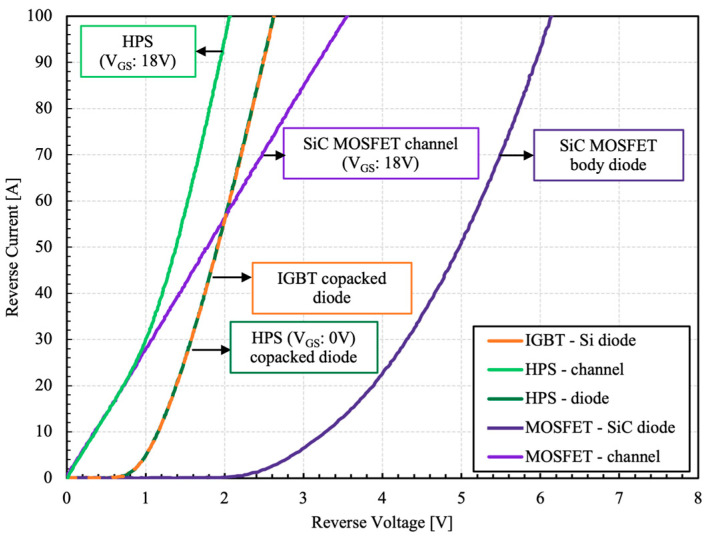
Measured reverse I–V characteristics of the HPS and its constituents at 25 °C [3].

One of the benefits of HPS is that reverse current flow is possible without the need of additional diodes. There are a number of paths for the current flow, which are presented in the reverse I–V characteristics of the HPS and its integral components, as shown in Figure 5.

The reverse current flow is permissible through the body diode or the channel of the MOSFET if a positive gate voltage larger than gate threshold is present. However, an additional anti-parallel diode may be necessary to meet higher current rating requirements. In this case, a Si soft recovery diode is employed. The diode activates at a high load current of above ~25 A, which takes additional load off the MOSFET channel. The SiC body diode does not contribute to any current flow under normal operating conditions due to its very high bipolar on-stet voltage.

#### 4.1.2. Capacitance Voltage Characteristics

**Figure 6:**
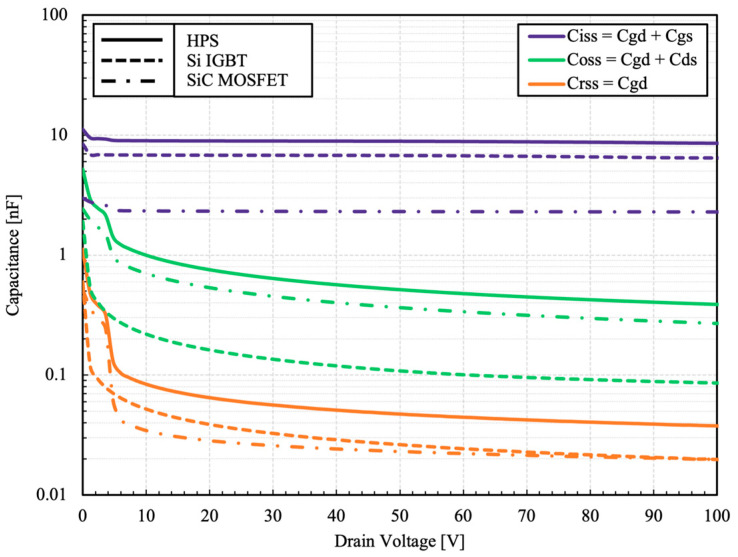
Measured capacitance as a function of drain voltage at 100 kHz. V_GS_ = 0 V.

The parasitic small signal capacitances of the HPS are presented in Figure 6.

The capacitance of the HPS equals to the sum of its constituent components. However, because of different switching sequences depending on the gate configuration, the effective capacitance of the HPS can be lower. In dual gate configuration, during the turn-off, the capacitances of the IGBT are charged in advance of the MOSFET. The output capacitance of the IGBT acts merely as a small parallel capacitance to the MOSFET during the switching transient. Therefore, the effective capacitances of the HPS at the instance of switching is almost identical to its constituent MOSFET, which ultimately determines the switching characteristic of the device. The same is true for the turn-on switching in reverse order.

### 4.2. Influence of Delay Time on Switching Characteristics

**Figure 7:**
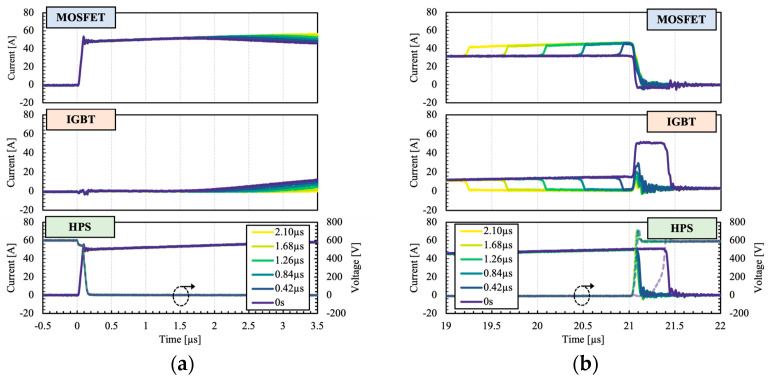
Measured (**a**) turn-on and (**b**) turn-off switching waveforms as a function of delay times.

As mentioned previously, the dual gate configuration of the HPS provides additional flexibility and control over the switching transitions. For optimal switching performance, a delay is necessary to ensure the MOSFET regulates the switching transitions. Figure 7 shows the influence of delay time on current sharing between the HPS constituents.

It can be observed that at 0 s delay, the IGBT turns-off after the MOSFET, which leads to higher switching losses and bipolar transition. When an appropriate delay is added, the IGBT can be turned off prior to the MOSFET, under a zero-voltage switching condition which effectively suppresses the associated IGBT switching losses. A secondary hump in the IGBT current waveforms can be seen after the turn-off transition (Figure 7b), attributed to the charging process of the IGBT parasitic output capacitance.

**Figure 8:**
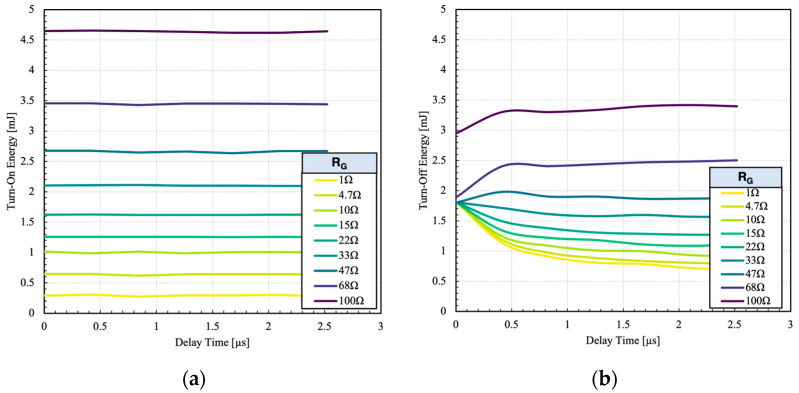
Influence of delay on (**a**) turn-on and (**b**) turn-off energy losses at different gate resistances.

The corresponding turn-on and turn-off switching energy as a function of delay time is shown in Figure 8.

At low gate resistances (R_G_ < 33 Ω), the turn-off losses decrease with longer delays. However, at higher gate resistances, the energy loss gets larger due to the fact the MOSFET is switching at lower dV/dt values. Contrary to the turn-off, the inherently longer delay of the IGBT eliminates the need for additional delay period for the turn-on. As the IGBT turns on, the MOSFET current starts to decrease and the excess current is diverted to the IGBT. Note that the current diversion is not instantaneous as the IGBT gate capacitances need to be fully charged. Because of the inherently longer delay of the IGBT, additional delay does not affect the turn-on energy loss, as can be seen in Figure 8a. However, the delay should be kept small to minimize the additional load on the MOSFET. In addition, the operating frequency of the HPS is limited by the imposed delay. This means that the HPS cannot switch at higher frequency than its constituents when handling the full load current. At low load current, however, it is possible to increase the frequency by completely turning-off the IGBT, eliminating the delay.

### 4.3. Switching Characteristics

#### 4.3.1. Influence of Gate Resistance on Switching

**Figure 9:**
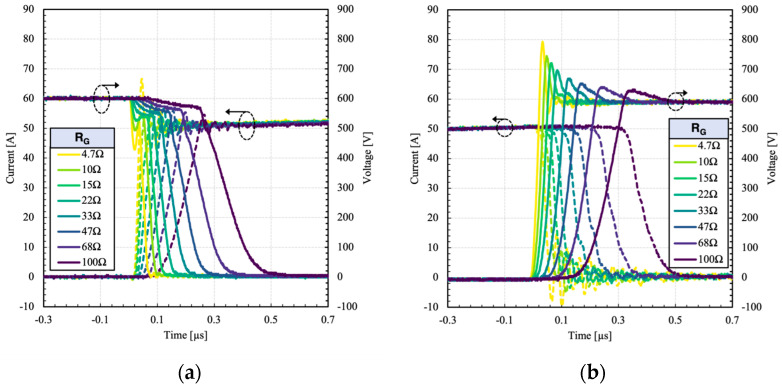
Measured (**a**) turn-on and (**b**) turn-off switching waveforms of the dual gate HPS at different gate resistances [3].

In dual gate configuration, the switching can be optimized by imposing a delay. In this case, a 1 µs delay is added during the turn-off, while the delay for the turn-on is set to 0 s. The switching waveforms of the dual gate HPS at different gate resistances (R_G2_) are presented in Figure 9.

Since the IGBT is switched off prior to the MOSFET, all the minority carriers are removed. Thus, no tail current can be observed during the turn-off. The surge voltage decreases with the gate resistance, as expected, due to slower dV/dt. It is important to point out that in standalone IGBTs, the surge voltage increases with the increase of gate resistance (under high dV/dt turn-off conditions) which indicates occurrence of dynamic avalanche (DA) as described in [3,17,18]. However, in HPS, dynamic avalanche is inherently suppressed because there is no high voltage built up across the IGBT (and dV/dt = 0) during its turn-off, and all its current is diverted to the MOSFET.

#### 4.3.2. dV/dt Controllability and Switching Losses

**Figure 10:**
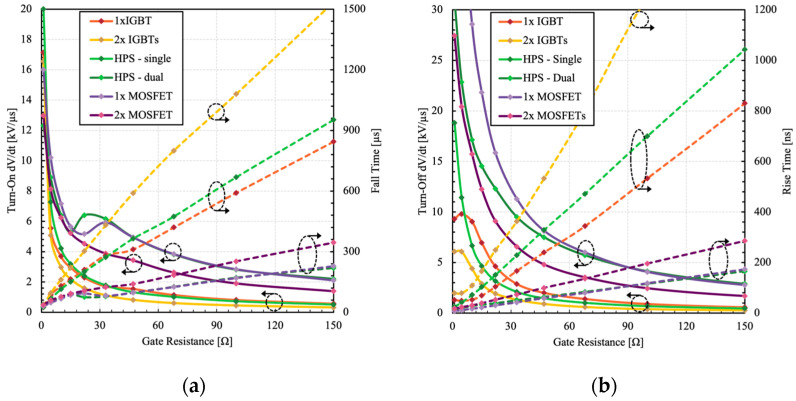
Measured (**a**) turn-on dV/dt and fall time and (**b**) turn-off dV/dt and rise time as a function of different gate resistances.

The turn-on and turn-off dV/dt and switching time were extracted from the measured switching data, as shown in Figure 10.

**Figure 11:**
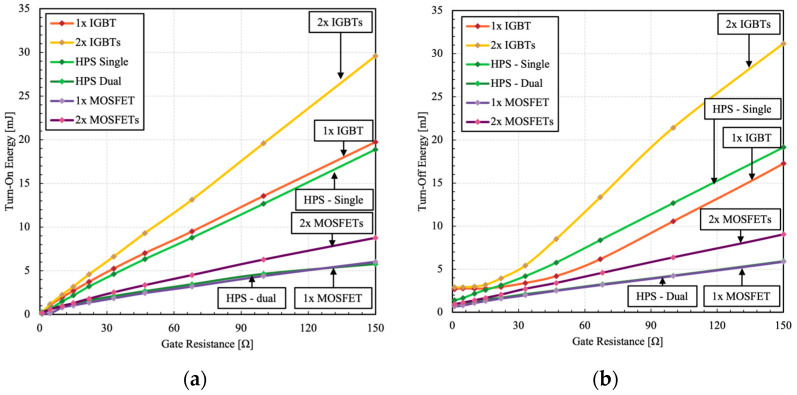
Measured (**a**) turn-on and (**b**) turn-off switching energy losses as a function of different gate resistances.

Depending on the application, the slew rate may need to be adjusted to meet the design requirement. For example, in motor control applications, high dV/dt can increase the stress on winding and insulations, which limits their useful lifespan [19,20,21]. Such systems conventionally utilize bulky filters to control and limit dV/dt and electromagnetic interference (EMI) based on the system requirements [22]. The HPS can operate over a wide range of dV/dt values depending on the gate resistance, thus meeting various motor drive application requirements. This also helps to manage EMI related issues. The corresponding dissipated switching energy is presented in Figure 11.

It can be observed that the switching losses of the HPS with dual gate control are virtually identical to its constituent MOSFET. This confirms that the switching transition is in unipolar mode and handled through the constituent MOSFET. On the other hand, the single gate configuration shows higher losses, though its magnitude is still significantly lower than that of 2× IGBTs. Additionally, the HPS shows lower switching losses than the equivalent 2× MOSFETs. Another observation is that in IGBTs, the turn-off losses do not decrease at low gate resistances due to DA effect. Such limitation is suppressed in the HPS, allowing its constituent IGBT to operate at higher dV/dt.

#### 4.3.3. Influence of Load Current on Switching

**Figure 12:**
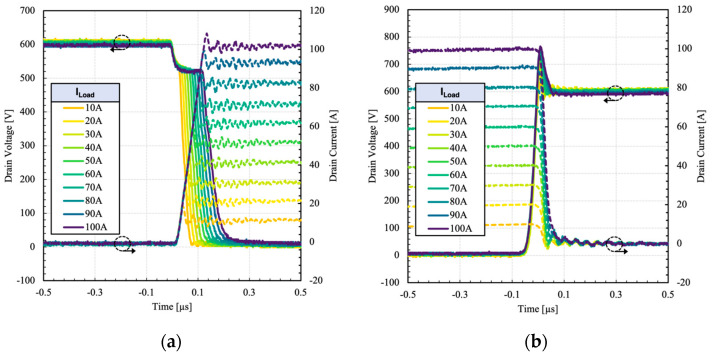
Measured (**a**) turn-on and (**b**) turn-off switching waveforms of the dual gate HPS at different load currents. R_G2_ = 22 Ω.

Figure 12 shows the switching waveforms of the HPS at various load currents, from 10 A to 100 A. The gate drive parameters are not changed from the previous section.

**Figure 13:**
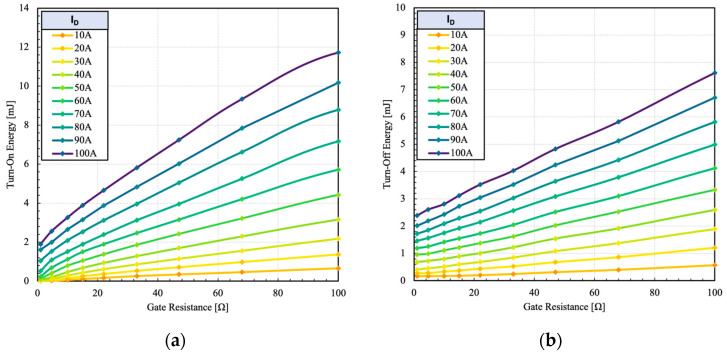
Measured (**a**) turn-on and (**b**) turn-off switching energy of the dual gate HPS at different load currents and 600 V.

The test was repeated at different gate resistances and current levels to determine switching loss characteristics, as shown in Figure 13.

Because of the negligible switching losses due to the constituent IGBT, the device can operate at higher currents. The HPS maintains a low switching loss profile even at higher current due to unipolar switching transitions.

### 4.4. Power Loss Contribution

**Figure 14:**
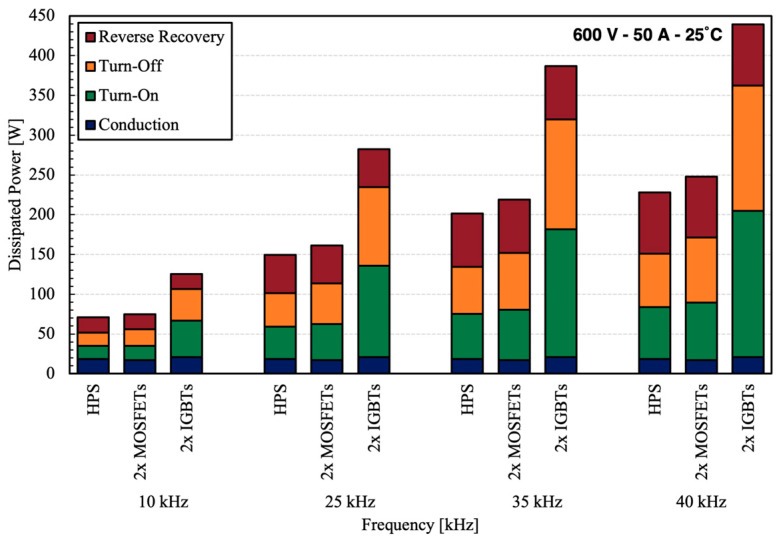
Comparison of dissipated power at different frequencies. The duty cycle is assumed to be 0.3 and the gate resistance R_G2_ is 22 Ω.

Figure 14 shows the contribution of conduction and switching losses to overall power loss associated with each device at different frequencies, based on the measurement data.

For this comparison, a turn-off delay of 1 µs is applied based on the data presented in Figure 8. The switching losses are frequency dependent and therefore increase as the frequency rises. The conduction loss corresponds to the on-state voltage drop and does not change with the frequency. The HPS shows the lowest overall loss compared to 2× MOSFETs and 2× IGBTs, indicating its greater performance. At higher frequencies, IGBTs are limited by the switching losses that prevent their use. If a Si PiN diode is used, the turn-on losses will be higher due to noticeably higher reverse recovery losses compared to those of SiC Schottky diodes. The reverse recovery loss associated with the Si diode is presented separately in Figure 14.

### 4.5. Short-Circuit Withstanding Capability

**Figure 15:**
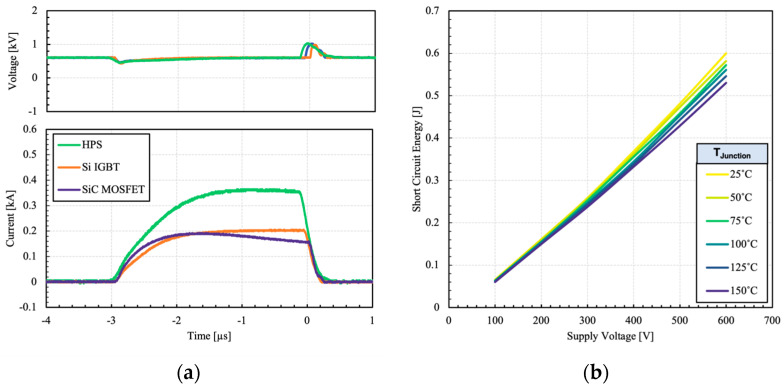
(**a**) Short-circuit waveforms the HPS and its constituents at 150 °C. (**b**) Dissipated energy during short-circuit event as a function of different bus voltages and different temperatures. V_GS_ is 15 V.

Short-circuit capability is a key measure of device reliability and ruggedness operating under fault conditions. Under short-circuit conditions, a massive current flows through the device that is several times higher than the rated current. While Si IGBTs have traditionally offered short-circuit capability time in the range of 10 µs, SiC devices have struggled to withstand short-circuit in a comparable manner. The temperature coefficient of saturation current in SiC devices depends on the gate voltage. At low gate voltage, it has been observed that the saturation current increases, while at high gate voltage, the saturation current decreases with temperature. This is because of the channel resistance, which has a negative temperature coefficient, is dominant at low gate voltages whereas the drift region resistance, which has a positive temperature coefficient, is dominant at high gate voltages [23]. In case of the HPS, the worst-case scenario occurs when its constituents are subject to fault for the same duration. Figure 15 shows the HPS switching waveforms and dissipated energy under short-circuit.

The short-circuit current is ultimately determined by the corresponding saturation current. It is clear that the short-circuit current of the HPS is equal to the sum of the MOSFET and IGBT currents. The surge voltage at the end is caused by stray inductances present in the power loop. The short-circuit current droop in the MOSFET is associated with self-heating effect as the carrier mobility decreases. While both devices are subjected to high short-circuit currents, due to smaller die area, the SiC device has a smaller heat capacity. Also, the on-state resistance and subsequently the saturation current of SiC are strongly influenced by the temperature. Therefore, it is subject to a higher degree of self-heating. The test was repeated at different supply voltages and temperatures. The dissipated energy during the short-circuit event was measured as shown in Figure 15b.

It can be observed that the dissipated energy increases linearly with the supply voltage. As the temperature rises, the dissipated energy decreases due to the positive temperature coefficient of saturation current, which results in a lower short-circuit current.

## 5. Conclusions

In this paper, a detailed analysis of a Si–SiC hybrid power switch has been presented in a comprehensive manner. The device characteristics, short-circuit capability, and switching performance were demonstrated experimentally. The HPS can benefit from the advantages of Si IGBT and SiC MOSFET technologies compared to Si or SiC standalone devices. The HPS can operate in either unipolar mode or bipolar mode depending on load current and temperature. This unique dual-mode operation enables a significant reduction in on-state losses over a wide range of loads. With intelligent control of the gates, the switching losses of the HPS can be lowered to as low as its integral MOSFET as a standalone device. The HPS can be scaled according to application requirements by addition of more IGBTs and MOSFETs to fit a variety of power systems. In conclusion, the HPS can offer an improved cost–performance ratio due to abundant supply of cheap Si devices alongside high performance SiC devices, which is well-suited for power module integration. Further research could evaluate different ratios of Si to SiC and how it affects the performance. Different control strategies can potentially improve the performance by turning the IGBT completely off under low load conditions. Also, different combinations of HPS based on emerging semiconductor technologies, such as GaN, can be of great interest to reduce losses even further.

## Data Availability

The original data is contained within the article. Any further inquiries can be directed to the corresponding author.
